# Bisbibenzyls, a New Type of Antifungal Agent, Inhibit Morphogenesis Switch and Biofilm Formation through Upregulation of *DPP3* in *Candida albicans*

**DOI:** 10.1371/journal.pone.0028953

**Published:** 2011-12-12

**Authors:** Li Zhang, Wenqiang Chang, Bin Sun, Matthias Groh, Andreas Speicher, Hongxiang Lou

**Affiliations:** 1 School of Pharmaceutical Sciences, Shandong University, Jinan, Shandong, China; 2 Chemistry – Organic Chemistry, Saarland University, Saarbrücken, Germany; Institute of Developmental Biology and Cancer Research, France

## Abstract

The yeast-to-hypha transition plays a crucial role in the pathogenesis of *C. albicans*. Farnesol, a quorum sensing molecule (QSM) secreted by the fungal itself, could prevent the formation of hyphae and subsequently lead to the defect of biofilm formation. The *DPP3*, encoding phosphatase, is a key gene in regulating farnesol synthesis. In this study, we screened 24 bisbibenzyls and 2 bibenzyls that were isolated from bryophytes or chemically synthesized by using CLSI method for antifungal effect. Seven bisbibenzyls were found to have antifungal effects with IC_80_ less than 32 µg/ml, and among them, plagiochin F, isoriccardin C and BS-34 were found to inhibit the hyphae and biofilm formation of *C. albicans* in a dose-dependent manner. To uncover the underlying relationship between morphogenesis switch and QSM formation, we measured the farnesol production by HPLC-MS and quantified Dpp3 expression by detecting the fluorescent intensity of green fluorescent protein tagged strain using Confocal Laser Scanning microscopy and Multifunction Microplate Reader. The *DPP3* transcripts were determined by real-time PCR. The data indicated that the bisbibenzyls exerted antifungal effects through stimulating the synthesis of farnesol *via* upregulation of Dpp3, suggesting a potential antifungal application of bisbibenzyls. In addition, our assay provides a novel, visual and convenient method to measure active compounds against morphogenesis switch.

## Introduction

*Candida albicans*, which can change from symbiotic form to pathogenic form upon an alteration in the external environment, causes shallow surface infections as well as deep infections [1]. In recent years, the over use of wide-spectrum antibiotics, long and repeated treatment have caused pathogenic fungi resistant to anti-fungal agents, especially to fungistatic drugs [2], and thereby more effective therapeutic drugs or alternative treatments are needed to alleviate the situation. Studies have demonstrated that filamentation plays a crucial role in the pathogenic process [3], [4], [5]. The transition from yeast to hyphae could improve the virulence of *C. albicans* during the course of infection [6]. Filaments generate strong top pressure for permeation into host body and have the advantage to escape from immune cells [7]. In addition, hyphae serving as the skeleton of biofilms play a critical role for the formation of highly heterogeneous architecture, which protects microorganisms from antibiotic treatment and also creates a source of persistent infection [7], [8], [9]. Therefore, prevention of the hyphae formation could be an effective means to reduce the biofilm formation and virulence in the pathogenesis of *C. albicans*.

Farnesol, a quorum sensing molecule (QSM) secreted by *C. albicans*, could inhibit yeast-to-hypha conversion and biofilm formation [10], [11], [12], [13]. Some antifungal agents could stimulate farnesol production through inhibiting sterol synthesis in fungi [14]. *DPP3*, encoding phosphatase, has been demonstrated to be involved in farnesol synthesis by converting farnesyl pyrophosphate to farnesol [15].

Bisbibenzyls, a group of phenolic compounds exclusively occurred in liverwort, have potent bioactivities serving as antifungal, antioxidant and antitumor compounds [16]. We have previously shown that plagiochin E (A6) [17] extracted from *Marchantia polymorpha* could reverse fungal resistance to fluconazole and induce apoptosis of *C. albicans*; Riccardin D (A4) [18] from *Dumortiera hirsute* inhibits hyphae formation and thereby interfere with the biofilm formation. In the present experiments, 24 bisbibenzyls and 2 bibenzyls extracted from liverwort plants or chemically synthesized were screened for antifungal activity. We found three compounds with good antifungal activities that displayed bioactivity against hyphae formation. We also measured Dpp3 expression using GFP-tagged *Candida albicans* strain (BWP17-*DPP3*-*GFP*) to investigate the underlying relationship between morphogenesis switch and QSM formation. We showed that PLF (A1), isoriccardin C (A3) and BS-34 (A5) inhibits hyphal and biofilm formation in a dose-dependent manner, which was consistent with the elevated farnesol production and Dpp3 expression. The findings suggest that the tested agents could inhibit yeast-to-hypha transition by stimulating farnesol production through upregulating Dpp3, and thus leading to the failure of biofilm formation.

## Methods

### Media and strains

*C. albicans* strains BWP17 [19], SC5314 [20], CA2 [21], CA10 [22] and YEM30 [23] were used in MIC_80_ detection, and the transformant of BWP17 (BWP17-*DPP3*-*GFP*) was used throughout the studies, stored at −80°C. The *C. albican* strain SC5314 and YEM30 are wild types, CA2 is a clinical azole-sensitive strain while CA10 is clinical azole-resistant [18]. BWP17-*DPP3*-*GFP* was constructed by transforming GFP into the strain of BWP17. The strains were grown in yeast extract peptone dextrose (YPD, 2% tryptone, 1% yeast extract, 2% glucose) solid medium containing 2% agar, and subcultured overnight to stationary phase with shaking at 30°C in YPD medium at least twice. Cells were harvested and washed twice in sterile water and re-suspended in RPMI 1640 medium plus morpholinepropanesulfonic acid (MOPS) and adjusted to the desired density after counting with a hematocytometer.

### Antifungal agents

**Figure 1:**
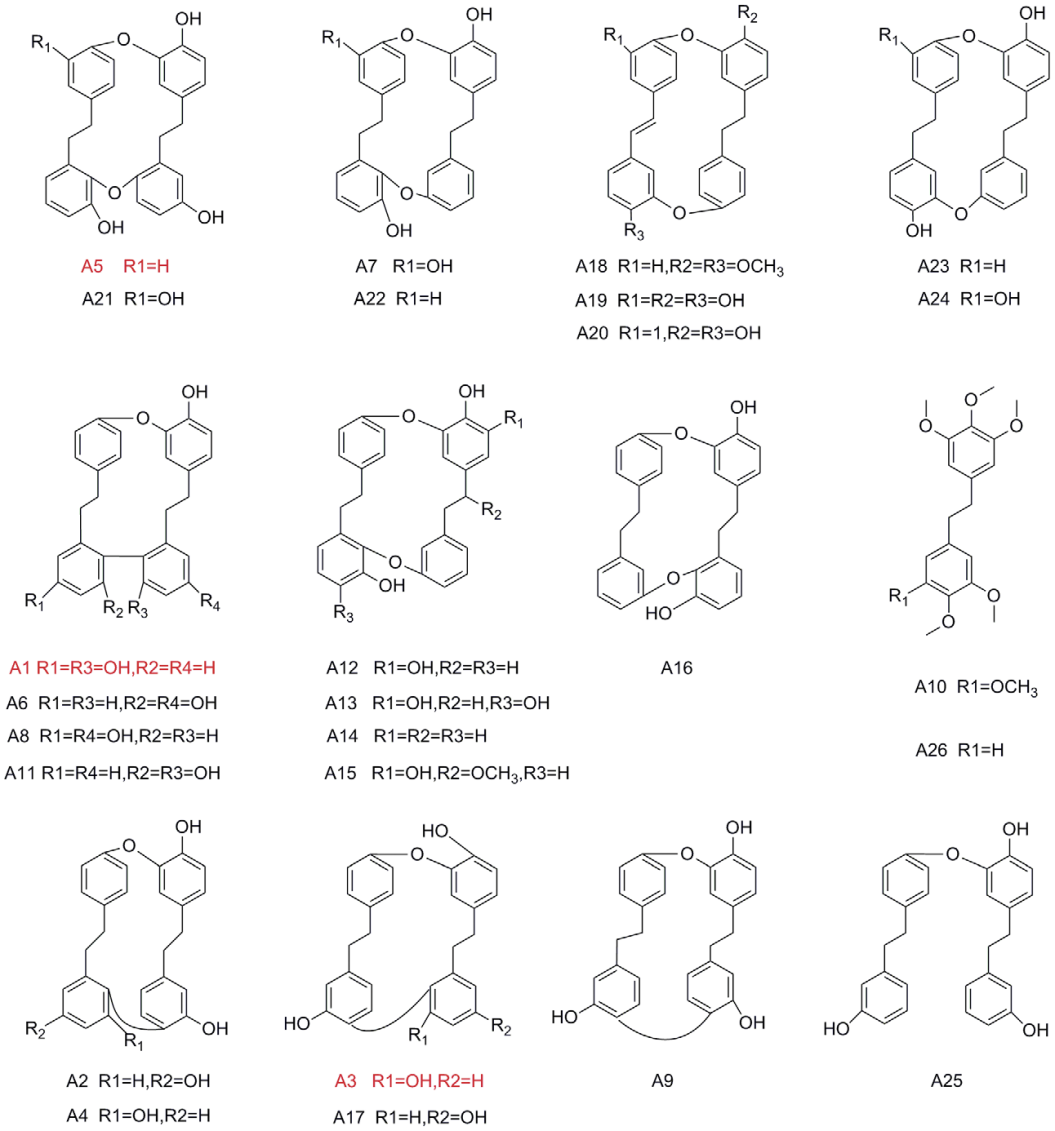
Chemical structures of bisbibenzyls tested in the antifungal screen. Marked in red were the ones used for further research.

The compounds were naturally isolated from liverwort in our laboratory except the synthesized compounds including A1, A6, plagiochin G (A8), polymorphatin A (A9), plagiochin H (A11) and isoriccardin D (A17) (Fig. 1). All the compounds were diluted with dimethylsulfoxide (DMSO) to 20480 µg/ml as stock solutions and stored at −20°C. Final desired concentration was diluted with RPMI 1640 medium.

### Construction of green fluorescent protein tagged strain

The *C. albicans DPP3*-*GFP* strain (MG1000) was created by homologous recombination of green fluorescent protein (GFP) sequences into the 3′ end of the *DPP3* open reading frame (ORF). The DNA used for the transformation was created by PCR using primers containing ∼70 base pairs of sequence homologous to the 3′ end of the *DPP3* open reading frame to amplify a cassette containing GFP and a URA3 selectable marker using pGFP-URA3 as the template [24]. The DPP3-GFP fragment was amplified using the primers (5′-TAAAAGGAAAGAAGAAATTAAACAAGATGAAAACAATTATAGAAGAATATCAGACATTTCCACTAATGTGGGTGGTGGTTCTAAAGGTGAAGAATTATT-3′ and 5′-GAAAAGTAAAGCCCAACCAGACAGACGACTAGACCAAGACTAGCCCTGGGGGGGAGCTAAGTAGTATCTAGAAGGACCACCTTTGATTG-3′). The PCR product was transformed into auxotrophic mutant strain BWP17. The transformed strain was then spotted on SD minus uracil (SD − Ura) solid medium and grown at 37°C for 3 d. The colonies resulting from the transformation were then screened for GFP-positive cells by fluorescent microscopy.

### Antifungal susceptibility study

The minimal inhibitory concentration (MIC) of the tested agents were determined by the broth microdilution procedure recommended by the Clinical and Laboratory Standards Institute (formerly the National Committee for Clinical Laboratory Standards) [25]. Cells were cultured in 96-wells plate at a density of (3–5)×10^3^ cells/ml. The yeast suspension was treated with the tested agents on several concentrations with two-fold increase, while the negative control was conducted with the same amount of vehicle. Testing of these compounds was performed in duplicate and incubated for 24 h at 35°C.

### Anti-hyphal and biolfilm inhibition test

The measurement of hyphal growth was carried out with a modified broth micro-dilution method [18], cells were diluted with RPMI 1640 to 10^5^ cells/ml, and then the tested agents were added into the yeast suspension to reach the final concentration ranging from 0–64 µg/ml with 2-fold increase. They were incubated at 37°C and photographed at indicated time. And the fluorescence images were captured with the same exposure time. The morphology of *C. albicans* cells was assessed directly by microscopy analysis. To detect hyphae formation on solid media [26], Spider (1% [wt/vol] nutrient broth, 1% [wt/vol] mannitol, 0.2% [wt/vol] K_2_HPO_4_, with pH 7.2) agar plates containing different concentrations of A1, A3 and A5 (Fig. 1) (ranging from 8 µg/ml to 64 µg/ml with 2-fold increase) were used to test morphology alteration of BWP17-*DPP3-GFP* cells, the images were captured from 2 d to 5 d using the 4× objectives of an inverted fluorescent microscope. The effect of identified agents on biofilm formation were performed as previously described [27]. The final volume of 150 µl of yeast suspension with initial cell density of 1×10^6^ cells/ml was seeded into microtiter plates with various concentrations (0, 4, 8, 16, 32 and 64 µg/ml) of the compounds. 48 hours later, the supernatant was discarded, the biofilm was washed with sterile water at least three times to remove the planktonic cells, and then subject to XTT reduction assay [28]. Testing of these compounds was performed in quadruplicate. Besides, a visualized way was utilized for biofilm detection. The supernatant of performed biofilms was discarded and 100 µl PBS plus 10 µl alamar blue was added into the well, and then the color change was photographed after 4–6 hours' incubation in dark.

### Measurement of farnesol using HPLC-MS

In each group, thirteen milliliters of yeast suspension with concentration of 10^5^ cells/ml, was treated with the tested agents at the final concentration of 2× MIC, 1× MIC, 0.5× MIC and 0 µg/ml, and incubated at 37°C with shaking. Cells were harvested after 12 hours. The cell debris was dried and weighed with an electronic balance. Farnesol was extracted as previously described [29] and detected using HPLC-MS (LCMS-2020, SHIMADZU). The detection was performed with absorbance at 210-nm and a 5-mm C18 reversed-phase column (4.6 by 250 mm) was utilized with a mobile phase of 4∶1 methanol-H_2_O eluted at 1 ml/min flow rate. In order to obtain the standard curve for figuring out the concentration of farnesol secreted by *C. albicans* cells, six farnesol standard samples with different concentrations were applied to receive the integral areas eluting at its retention time. The farnesol extracted from the supernatant of each sample was calculated based on the HPLC integral area compared with standard curve.

### Detection of Dpp3 production using Confocal Laser Scanning microscopy and Multifunctional Microplate Reader

The Dpp3 expression was measured by detecting the fluorescent intensity of BWP17-*DPP3*-*GFP* strain with Confocal Laser Scanning microscopy (CLSM). *C. albicans* cells were cultured at 37°C and treated with different concentrations of the tested agents or fluconazole (FLC) (positive control). 12 hours later, the images were captured by CLSM and the fluorescence of GFP was excited by the laser of 488 nm with emission of 500–560 nm. The fluorescent intensity of the sample was also measured using a Multifunctional Microplate Reader (Berthold Biotechnologies, Bad Wildbad, Germany). Here the GFP was used as a marker for Dpp3 expression.

### *DPP3* expression detected by Real-Time Reverse Transcription Polymerase Chain Reaction (RT-PCR)

BWP17-*DPP3*-*GFP* cells were adjusted to 5×10^6^ cells/ml with RPMI 1640 medium, 5 ml of cells were incubated with A1 and A3 (32 µg/ml, 16 µg/ml and 8 µg/ml) and A5 (64 µg/ml, 32 µg/ml and 16 µg/ml) with vehicle as control at 37°C for 12, 24 and 48 hours. The total RNAs were isolated as previously described [30]. *GSP1* was used as the internal control. Measurement of the relative quantitative expression of *DPP3* levels was conducted through using an Eppendorf Mastercycler Real Time PCR System. And the gene relative expression was calculated using the formula 2^−ΔΔCT^.

### Statistical analysis

Values are mean±standard deviation (SD). Statistical significances were determined by Student's *t*-test and statistical significance was determined by *p*<0.05.

## Results

### In vitro antifungal activity

MIC_80_ test in RPMI 1640 medium with BWP17-*DPP3*-*GFP* strain showed that A1–A5 were 16 µg/ml, A6 and A7 were 32 µg/ml, A8–A10 were 64 µg/ml, respectively and the remaining were all greater than 64 µg/ml. Three (A1, A3 and A5) of them were selected to detect the MIC_80_ using other four *C. albicans* strains (Table 1) for further study.

**Table 1:** MIC_80_ of Biol Pharm Bull bisbibenzyls in different *Candida albicans* strains.

Strains	MIC_80_ (µg/ml)		
	A1	A3	A5
BWP17-*DPP3*-*GFP*	16	16	16
SC5314	16	16	16
CA2	16	32	32
CA10	16	16	16
YEM30	16	16	16

### Antifungal activity of mycelium growth and biofilm formation

**Figure 2:**
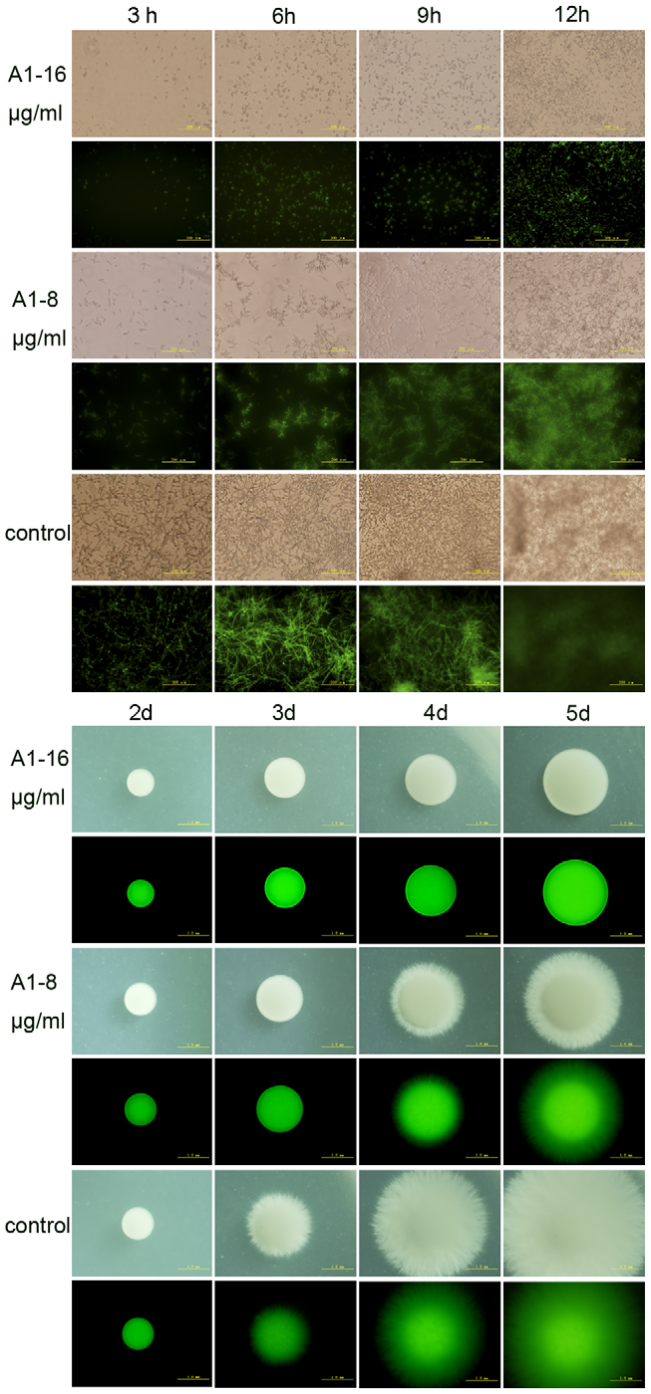
Phase-contrast micrographs of BWP-*DPP3-GFP* cells challenged by bisbibenzyls compounds within 12 hours. A) *Candida albicans* cells grown in RPMI 1640 broth medium was captured using an Olympus fluorescent microscope. The results showed that cells co-incubated with A1 (16 µg/ml) displayed yeast cells, cells with no drugs formed mycelium after three hours and maintained substantial growth of hyphae. B) Cells grown on Spider solid media were photographed from 2 d to 5 d using 4× objectives of an Olympus fluorescent microscope.

We next evaluated the activity of A1, A3 and A5 against morphological conversion and biofilm formation of *C. albicans*. Cells of BWP17-*DPP3*-*GFP* were cultured in RPMI 1640 medium or Spider agar plates containing gradient concentrations of agents at 37°C and then observed at indicated time under fluorescent microscope (Olympus IX71, Olympus, Tokyo, Japan). A1, A3 and A5 at the concentration of 16 µg/ml, completely inhibited hyphal formation in liquid RPMI 1640 medium within 12 hours and on Spider agar plates within 5 days (Fig. 2). The inhibitory effects were shown in a dose-dependent manner. A1 was chosen as an example to show the fungal morphological change as the time elapses. During 12 hours in liquid RPMI 1640 medium, cells were treated with 16 µg/ml of A1 maintained the yeast form with only a slight increase in the number. A suboptimal concentration of A1 (8 µg/ml) inhibited the hyphae growth but had no effect on germ tube formation. By contrast, cells in the negative control group formed the true hyphae after three hours and the filaments formed a criss-cross network architecture after 9 hours (Fig. 2 A). On Spider agar plates, the morphology of the colony was photographed from 2 to 5 days using 4× objective of an inverted fluorescent microscope. A1 at its MIC could block the yeast-to-hypha transition revealed by the colony that showed a very smooth round plaque without mycelium around the edges throughout the five days. When the dose of A1 was reduced to half of its MIC, the hyphae growth could only be partially inhibited. However, the negative control formed hyphae within 3 days (Fig. 2 B). Collectively, the data suggested the compounds could completely block the morphogenesis switch at the concentration of one MIC, and reduce the hyphae growth at half of the MICs.

**Figure 3:**
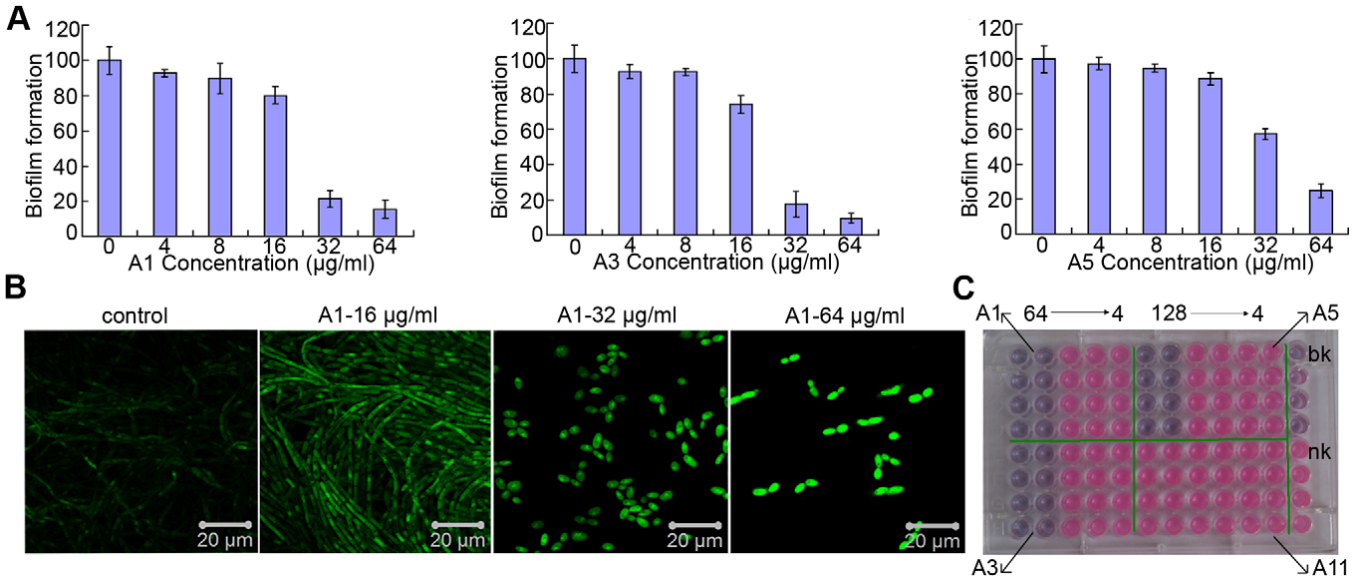
Biofilm formed of BWP17-*DPP3-GFP* cells pretreated with bisbibenzyls compounds. A) Quantitative measurement of inhibition of biofilm formation with Microplate Reader. B) Confocal micrographs of BWP17-*DPP3-GFP* cells cultured in RPMI 1640 for 48 hours. C) Cells were stained with alamar blue and cultured for 4–6 hours in dark, the supernatant would become pink when cells grow into biofilm, otherwise turn into blue. The test was performed in quadruplicate. A11 represents the compounds that biofilm inhibitory activity >64 µg/ml, bk represents blank control and nk represents negative control.

Biofilm formed by BWP17-*DPP3*-*GFP* was monitored by CLSM and its metabolism activity was evaluated using XTT reduction assay. The XTT results showed the biofilm inhibitory effect displayed a dose-dependent manner in the presence of detected agents. A1 and A3 reduced biofilm formation by about eighty percent at 32 µg/ml. And the biofilm mass was reduced to sixty percent by A5 at 32 µg/ml, approximately thirty percent at 64 µg/ml (Fig. 3A). CLSM analysis showed that cells within treated biofilm displayed yeast morphology when the concentration of A1 was 32 µg/ml or above. When the dose of A1 was reduced to 16 µg/ml or in control group, the biofilm predominantly composed of hyphae was formed (Fig. 3 B). We also utilized an alamar blue assay to evaluate the efficacy of compounds that inhibit the biofilm formation. The assay supported the conclusion that A1 (32 µg/ml or above) inhibited the biofilm formation, demonstrated by the blue color (Fig. 3 C). When the organism increased, the color changed from blue to pink as shown in low doses of A1 treated or negative control groups (Fig. 3 C).

### Enhancement of farnesol production by the tested compounds

**Figure 4:**
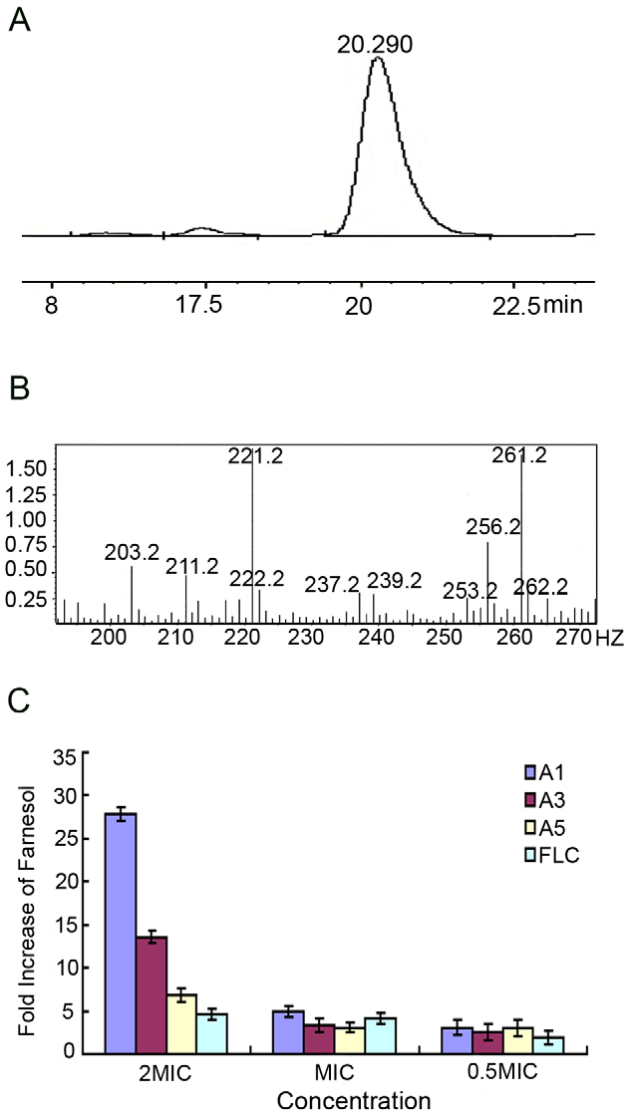
Farnesol production of BWP17-*DPP3-GFP* cells stimulated by bisbibenzyls. The cells were pretreated with A1, A3, A5 and FLC at 37°C for 12 hours and the farnesol amounts were determined by HPLC-MS. A) The retention time of farnesol eluting at 20.29 min. B) MS profile showed a molecular ion of m/z 221.2 and 222.2. C) Values of ordinate represents the increased folds of farnesol treated by tested agents compared with control.

To uncover the underlying mechanism of the tested agents in regulating the morphogenesis, we examined farnesol secreted by *C. albicans* by using HPLC-MS. Cells treated with the tested agents showed a higher amount of extracellular farnesol. The retention time of farnesol was at 20.29 min (Fig. 4 A). The peak eluting at 20.29 min was subjected to MS and showed a molecular ion of m/z 221.2 and 222.2, which further confirmed the evidence of farnesol (Fig. 4 B). The farnesol production increased as the dose of agents rose (Fig. 4 C). At 2× MIC, A1, A3, A5 stimulated farnesol production by 27.9, 12.7 and 6.9-fold increases, respectively, compared with that in negative control. The farnesol production showed 5, 3.3, and 3.1-fold increase at 1× MIC, and 3.1, 3, and 2.6 fold-increase at 0.5× MIC, respectively. The positive control (FLC) could only promote the production of farnesol by 4.6, 4.2 and 1.9-fold respectively, at 2× MIC, 1× MIC and 0.5× MIC (Fig. 4 C).

### Dpp3 and *DPP3* expression induced by the tested agents

**Figure 5:**
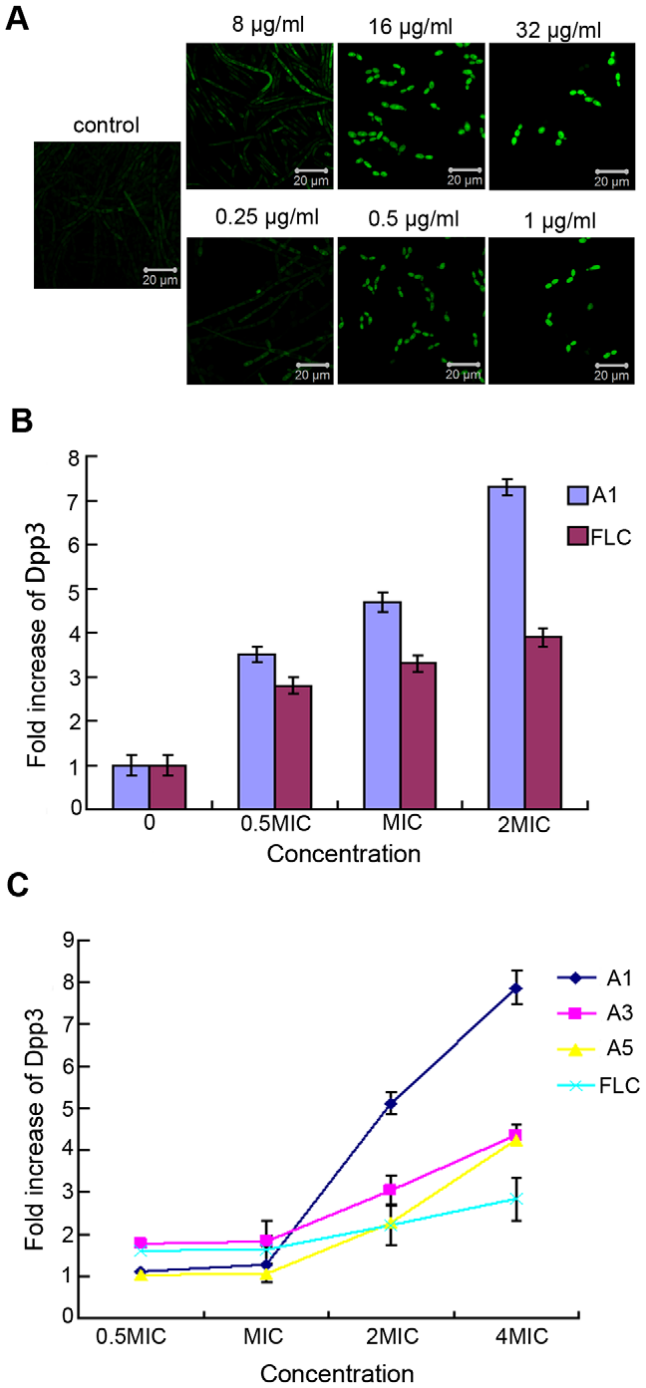
Dpp3 expression levels of BWP17-*DPP3-GFP* cells induced by bisbibenzyls. Values represent the increased folds of Dpp3 treated by tested agents compared with control. A) and B) Cells were treated with A1 and FLC at 37°C for 12 hours and then observed by CLSM and the Dpp3 expresson was quantitatively measured based on the fluorescence intensity. C) Fluorescence intensity of *C.albicans* cells pretreated with the tested agents was measured using a Multifunctional Microplater Reader after biofilm formation.

**Figure 6:**
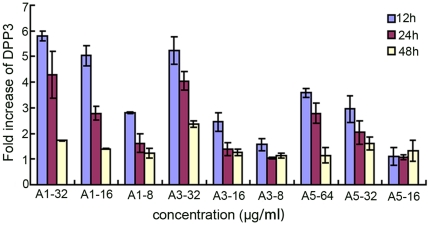
*DPP3* expression levels of BWP17-*DPP3-GFP* cells challenged with bisbibenzyls.

Given the results of farnesol production, we next utilized BWP17-*DPP3*-*GFP* strain to detect the expression of Dpp3, an enzyme converting farnesyl pyrophosphate to farnesol. The fluorescent intensity of GFP, as the indicator of Dpp3 expression, was measured by CLSM as well as Multifunctional Microplate Reader. CLSM analysis suggested Dpp3 expression displayed a dose-dependent manner under the treatment of tested agents after the cells were incubated for 12 hours at 37°C. The Dpp3 expression was augumented by A1 with almost 3.7-fold at 8 µg/ml, 4.7-fold at 16 µg/ml and 7.3 –fold at 32 µg/ml compared with control (Fig. 5 A, B). The results obtained from Multifunctional Microplate Reader also showed that the tested agents promoted the expression of Dpp3 in biofilm. Fluorescent intensity of cells exposed to A1, A3 and A5 from 64 µg/ml to 8 µg/ml showed a downward trend, while the biofilm formation changed from scattered form to mature one, suggesting that Dpp3 expression was negatively correlated with biofilm formation (Fig. 5 C). To determine whether the agents regulate Dpp3 in transcriptional level, we measured *DPP3* expression with real-time PCR and showed *DPP3* mRNA could be induced by the tested agents when the cells were cultured for 12 to 48 h, suggesting the antifungal activity of the tested compounds by upregulation of *DPP3* expression (Fig. 6).

## Discussion

As the earliest land plants, bryophytes grow in an unfavorable environment and inevitably produce secondary metabolites against different surviving stresses [31]. Among those diverse suite of fungi interacts with bryophytes as pathogens, which exhibit different methods of host cell disruption such as invading the host cell by hyphae formation or causing host protoplast degeneration [32]. Previous studies suggest these plants could biosynthesize some chemicals to control the plant diseases caused by fungi or bacteria [1].

*C. albicans*, as a pathogenic fungal, switches from yeast morphotype to filaments and develop the formation of biofilm to colonize in the host. Based on the aforementioned property of bryophytes, we evaluated the antifungal activity of their extracts. Our lab previously reported that bisbibenzyls including plagiochin E and riccardin D derived from bryophytes displayed a moderate antifungal action [33], [34]. We presume bisbibenzyls are the active agents in bryophytes to combat fungal invasion. In our present study, 26 bisbibenzyls isolated from liverworts and chemical synthesizes were screened for antifungal activities. Among them three compounds (A1, A3 and A5) showed a good effect in inhibiting the transition of yeast-to-hypha and biofilm formation (Fig. 2, 3). The effects of these compounds against biofilm formation were observed at or above their MICs. And they were the combination result of inhibitory growth and retarded the yeast to hyphal transition. Farnesol, a QSM, was found to regulate the morphogenesis switch and the inhibitory effect was positively correlated with farnesol formation as detected by HPLC-MS (Fig. 4). When the farnesol production was divided by the volume of culture, the concentration of farnesol could reach to 2.44–14.38 µM. At these concentrations, exogenous added farnesol could partially inhibit the hyphae formation or growth of *C. albicans* [13], but the inhibitory effect is less than that displayed by the tested agents. We speculated that it might be attributed to the better action of endogenous farnesol. *DPP3* encodes phosphatase which converts farnesyl pyrophosphate to farnesol [15]. To determine whether the agents stimulated farnesol production by upregulation of Dpp3, *C. albicans* strain BWP-*DPP3*-*GFP* was constructed. The results obtained from CLSM and a Multifunctional Microplate Reader showed Dpp3 was stimulated by the above agents, which was in accordance with the enhanced farnesol production (Fig. 5). To further investigate whether the upregulation of Dpp3 was the result of *DPP3* variation in transcript level, real-time PCR was performed. The results suggested that *DPP3* expression of *C. albicans* cells was promoted by the tested agents throughout the 48 h culture (Fig. 6), Based on above analysis, we concluded that the above agents induced the expression of *DPP3* encoding Dpp3, which synthesized more farnesol, to regulate hyphae and biofilm formation.

The clinical used antifungal agents were discovered based on killing the cells, at least inhibiting the growth. Azoles displayed fungistatic action by reducing the sterol synthesis; Echinocandins inhibit β-glucan synthase to synthesize the cell wall component and then kill the fungal cells; Rapamycin exerts its antifungal action by targeting the kinase Tor; Icofungipen functions as tRNA synthetase inhibitor to inhibit the cell growth [35]. However, agents targeting the virulence were less developed, although related genes of virulence were well investigated [36]. In this study, we investigated the application of bisbibenzyls against fungal invasion through regulating the known molecular mechanism of morphogenesis switch. Moreover, the structure of each active chemical agent gives a clue for further modification.

Taken together, we provided an alternative way to combat pathogenic fungi infection by targeting the step of morphogenesis switch. In addition, an effective assay for screening potential antifungal agents targeting farnesol synthesis is established through evaluating hyphal and biofilm formation by measuring Dpp3 expression and farnesol production.
